# Effects of working part-time and full-time on depression in old age

**DOI:** 10.21203/rs.3.rs-10396569/v1

**Published:** 2026-08-06

**Authors:** Tunga Kantarcı, Ingo Kolodziej, Bastiaan Froeling

**Affiliations:** 1 University of Groningen.; 2 Leibniz Institute for Economic Research (RWI) and Hochschule Fresenius University of Applied Sciences.; 3 University of Groningen.

**Keywords:** I10, J01, J22, J26

## Abstract

As retirement ages rise, many older adults are expected to work longer. However, not everyone can or wants to remain in full-time employment, making partial retirement an increasingly important pathway. This raises the question of how part-time work affects health, relative to both full-time work and full retirement. We analyze the mental-health outcomes of older adults in Europe, distinguishing between part-time workers, full-time workers, and retirees. After controlling for unobserved individual heterogeneity, we find that part-time employment deteriorates mental health to the same extent as full-time employment. Our findings suggest that reducing working hours at older ages does not provide additional mental-health benefits, whereas retirement appears to decrease depressive symptoms.

## 1. Introduction

Working becomes more common than ever before among older people, mainly due to rising statutory retirement ages. With increasing health care costs, understanding the implications of working and working conditions on health status in old age becomes vital. This is especially relevant given the rising number of people with mental health disorders. The World Health Organization (WHO) estimates that more than 1 billion people are living with mental health disorders, implying sizeable amounts of direct and indirect costs that amount to 1 trillion US dollars per year from depression and anxiety alone – whilst a large fraction of those affected does not receive adequate care (WHO, 2025). About one in six people living in the WHO European Region have a mental disorder (The Lancet Regional Health-Europe, 2025). Approximately 3.7% of Europeans live with anxiety disorders and 4.1% with depressive disorders (WHO, 2025). Excess costs of depression vary by socioeconomic factors, and indirect costs from sick leave and early retirement exceed direct costs from inpatient care, outpatient care and medication (König et al. 2021). Zhang et al. (2026) project that the global prevalence of depressive disorders will rise from 332.4 million cases in 2021 to over 466 million by 2040 – an increase of over 40 percent. From 2025 to 2050, anxiety, depression, and alcohol use disorders are projected to account for approximately 6% of annual healthcare expenditure across the 27 EU Member States, as well as Iceland, Norway, and Switzerland (OECD, 2026).

Research on the effects of retirement on physical and mental health draws mixed conclusions. Several studies suggest a positive effect of retirement on overall health (Insler, 2014; Coe and Zamarro, 2011; Eibich, 2015) and mental health (Charles, 2004; Eibich, 2015). Other studies find that retirement leads to worse cognitive functioning (Rohwedder and Willis, 2010; Bonsang et al., 2012) and an accelerated decline in cognitive abilities (Mazzonna and Peracchi, 2012). While Coe and Zamarro do not find an effect neither on cognitive abilities nor on mental health, Kolodziej and García-Gómez (2019) find protective effects of retirement on mental health with larger effects among those just around clinically defined thresholds of being at risk of depression, women, and blue-collar workers. In line with Kolodziej and García-Gómez, Barschkett et al. (2021) find that the increase in retirement age negatively affects health, including mental health. A meta study by Odone et al. (2021) supports these findings. An exception is Ebeid and Oguzoglu (2023) who find an increase in depression due to retirement.

Previous literature has mainly focussed on the effect of retirement against working any number of hours and did not distinguish between part-time and full-time work activity. Working part-time or full-time, rather than fully retiring, aligns with the principles of continuity theory by helping individuals maintain daily routines, preserve behavioral patterns, and sustain engagement in meaningful activities. Continued participation in the labour market also supports social connectedness and reinforces work-related role identity (Atchley, 1989; Ashforth et al., 2008).

This work role identity is likely to differ by the number of hours worked. Working part-time or full-time in comparison to working zero hours after retirement can affect mental health in old age differently. Individuals are likely to have different health-producing activities depending on the hours worked: working a distinctive number of hours differently affects the incentives and the time spent for building and maintaining (work related) human-capital and may operate through several mechanisms.

Differences in work hours can result in different forms of social interactions leading either to social isolation or an increase in social activities due to fewer time constraints. Social interactions, including type of social support and network participation, seem to be strongly associated with physical and mental health of the elderly (Cohen, 2004; Fiori et al., 2006; Litwin and Shiovitz-Ezra, 2011) and more specifically with depression (Schwarzbach et al., 2014). Moreover, the type of social network is likely to have an impact on health behaviour (Shiovitz-Ezra and Litwin, 2012). If social networks at work are important, then fewer hours spent at work means less frequent social interactions. Working fewer hours decreases the opportunity costs for a more active lifestyle in terms of time constraints as a key mechanism for mental health effects (Eibich, 2015).

There is limited evidence on how working part-time in old age influences health. For the US, Dave et al. (2008) find that individuals that are partially retired seem to have better mental health outcomes than individuals that are fully retired, but both groups are worse off compared to those who work full-time.

In this paper we analyse how full-time and part-time employment affect depression symptoms relative to retirement in Europe. We find that part-time work has an effect of similar magnitude to full-time work, indicating that reducing working hours does not provide additional mental-health benefits. Overall, the estimated effects are modest in absolute size. Compared with retirement, depressive-symptom scores rise by 0.115 points for part-time workers and by 0.135 points for full-time workers.

This finding relates to several strands of literature. First, partial retirement schemes are considered as an instrument to increase labour participation among older individuals. If older individuals more often work part-time instead of retiring due to promoting partial retirement schemes, partial retirement can increase total labour supply (Kantarcı et al., 2025). This makes it important to understand how part-time work, relative to full retirement, affects health and well-being in later life.

Second, a growing body of research highlights the rising prevalence of depressive symptoms and diagnoses among older adults and the substantial direct and indirect costs associated with them (Chatterji et al., 2007; Van den Berg and Ferrer-i-Carbonell, 2007). This has intensified interest in identifying work-related determinants of mental health in later life (Belloni et al., 2022; Calvo et al., 2013; Coe and Zamarro, 2011).

Third, the large literature on the retirement effects of physical and mental health documents both beneficial and adverse effects depending on context, population, and outcome measures. We contribute to this literature by providing new evidence on how adjustments in working hours, specifically, part-time versus full-time work, affect mental health, by comparing these effects directly to those of full retirement.

The reminder of this paper proceeds as follows. Section 2 discusses the empirical model. Section 3 describes the data as well as the health and work effort indicators, and Section 4 explores the data using graphical analysis. Section 5 presents the results. Section 6 concludes.

## 2. Empirical approach

Our aim is to analyse the causal effect of working part-time and full-time on health. We define two dummy variables, P_it_ and F_it_, indicating whether respondent i works part-time or full-time for individual i, at time t. Using OLS, we can estimate the health effects of working different numbers of hours using the following specification:

(1) $${\text{Y}}_{\text{it}}=\text{a}+\text{β}\cdot {\text{W}}_{\text{it}}+\text{f}\left({\text{A}}_{\text{it}}\right)+{\text{u}}_{\text{it}}$$

where Y_it_ is a measure of health, α is a constant term, and u_it_ is the error term. The vector W_it_ contains the two work-status indicators, P_it_ and F_it_. The coefficient vector β captures the effect of working part-time and full-time on health relative to the omitted category retirement. The function f(A_it_) controls for a function of age, as health is expected to deteriorate with increasing age linearly or non-linearly.

For equation (1) to yield consistent estimates of β, strict exogeneity must hold: $E\left[u_{it}|\right.$ $W_{\mathrm{it}}=0$. This assumption is potentially violated because there may be individual-specific, time-invariant characteristics absorbed in u_it_ that are correlated with both health Y_it_ and the decision to work. For example, as a job characteristic, occupation type may lead to a biased estimate: individuals in physically demanding jobs may experience poorer health, and this deterioration may in turn influence their labour supply decisions. To account for such unobserved, time-invariant heterogeneity, we augment equation (1) by including individual fixed effects:

(2) $${\text{Y}}_{\text{it}}=\text{α}+\text{β}\cdot {\text{W}}_{\text{it}}+\text{f}\left({\text{A}}_{\text{it}}\right)+{\text{e}}_{\text{i}}+{\text{v}}_{\text{it}}$$

The term e_i_ captures time-invariant individual characteristics, allowing us to obtain consistent estimates of β under the weaker assumption that $E\left[v_{it}|W_{it},e_{i}\right]=0$.

In this model, however, v_it_ may still be endogenous due to simultaneity: health conditions themselves can influence labour supply decisions. For example, Cai and Kalb (2006) show that, in Australia, better health increases the likelihood of labour force participation, particularly among older individuals. Lu et al. (2009) find that in China individuals with declining mental health are less likely to work. Similarly, Mols et al. (2012) report that almost one-third of cancer survivors in the Netherlands adjust their working situation after diagnosis, with most switching to part-time work or leaving employment altogether.

To address potential endogeneity arising from simultaneity, we consider taking an instrumental-variables (IV) approach. However, in our IV analysis, we do not find statistical evidence that mental health is endogenous to part-time and full-time work decisions (see appendix A.3). Therefore, we do not pursue the IV strategy in our main analysis. Consequently, we base our analysis on the model given by equation (2).

## 3. Data

To study the effect of working on depression, we use data from the Survey of Health, Ageing and Retirement in Europe (SHARE). We utilize data on 12 of the countries included in SHARE, focusing on health variables and work-related variables. From the SHARE dataset we use information from waves 1 through 8, except for wave 3, since this wave does not contain the same health variables as the other waves. The production dataset contains information from 2004 up to 2020 for people aged 50 and over.

We impose several sample restrictions. First, we drop an observation if the respondent reported to not have done paid work in any of the survey waves. This is because we want to determine the effect of working on health for which we need a respondent to work at some point in the survey. Second, we drop respondents who state to be ‘employed’, ‘unemployed’, ‘disabled’, ‘homemaker’ or ‘other’ after reporting that they were retired in any of the previous waves. This ensures that retirement is an absorbing state. Third, an observation is dropped if the respondent is unemployed, disabled, homemaker or other in any of the survey waves. These respondents work 0 hours, like retirees, but they are likely more active as they are not earning wages from working or receiving income from their pension. Fourth, an observation is dropped if the respondent did not do any work since age 50, i.e. if the last job ended before age 50. Finally, we drop observations of respondents who were younger than 50 or older than 70 years of age to focus on the years where respondents changed from working to retirement.

These sample restrictions lead to an unbalanced panel of 114,277 observations for 42,568 individuals. These observations are for the following countries: Austria, Germany, Sweden, The Netherlands, Spain, Italy, France, Denmark, Greece, Switzerland, Belgium and the Czech Republic, which are included in all waves.

### Depression score

We use the EURO-D scale which measures current depression and is constructed from several questions as a composite index of 12 items: depressed mood, pessimism, suicidality, guilt, sleep, interest, irritability, appetite, fatigue, concentration, enjoyment, and tearfulness. The scale ranges from 0, ‘not depressed’, to 12, ‘very depressed’. The scale has a cut-off point of depression at indicating at least four depressive symptoms (Prince et al., 1999). This measure has been validated and widely used for comparison of prevalence and risk of depression in cross country studies, including particular focus of occupational settings (e.g. Castro-Costa et al.; 2008, Larraga et al., 2006; Prince et al., 1999; Kolodziej and García-Gómez, 2019; Tomás et al., 2022).

### Number of work hours

Part-time or full-time work can be defined in several ways. Earnings, the number of hours worked per week, or the number of months worked per year are possible indicators of work effort. We base our definitions of part-time and full-time work on both the number of hours and months worked by a respondent. In the SHARE survey, data on these two measures are collected by asking respondents, “How many hours a week do you usually work in this job, excluding meal breaks (but including any paid or unpaid overtime)?” and “How many months a year are you normally working in this job (including paid holidays)?”.

**Figure 1:**
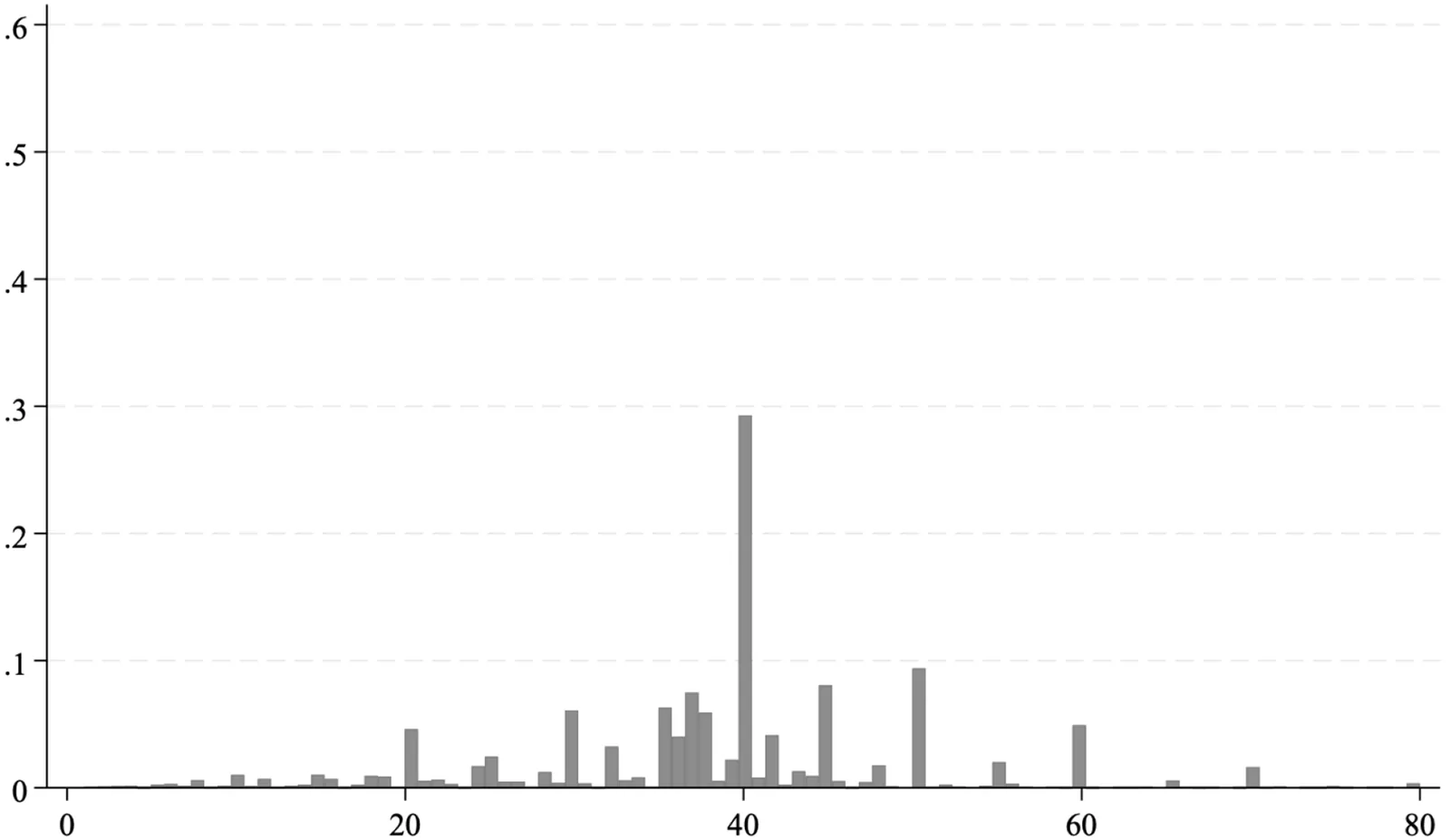
Distribution of the number of hours worked across all selected countries.

Figure 1 presents the distribution of the number of hours worked per week in the main job when data from all countries and survey years are combined. The most frequent number of hours worked per week is 40 in most of the countries, but attains a minimum of 35 hours in France, and 37, 38, and 42 in Denmark, Belgium, and Switzerland, respectively. These numbers define the number of hours spent in a full-time job during a week in the respective countries. In the whole sample, among those working less than 35 hours a week, 58.4% are working 21 or more hours a week. Considering the number of months worked in a year, in all countries the vast majority (97%) is working at least 11 months in a year. The number of months respondents spend working during a year has little variation across or within the countries. This suggests that our results should remain robust to different choices of threshold numbers of months worked per year in distinguishing between part-time and full-time work.

Since there is some heterogeneity in the most frequent number of hours worked across the countries, we adopt country-specific threshold numbers of hours worked per week to distinguish part-time from full-time employment, and define them accordingly. For all countries except France, Denmark, Belgium and Switzerland, we define full-time work as working 35 or more hours a week for 8 months or more in a year. We define part-time work as working less than 35 hours a week for 8 months or more a year, or as working 35 or more hours a week but less than 8 months a year. We define retirement as working 0 hours a week. The hours and months of work from both the main and a possible second job are considered in determining whether a respondent is working part-time or full-time. For France, Denmark, Belgium and Switzerland, we define full-time work as working 30, 32, 33, and 37 or more hours a week, respectively, for 8 months or more in a year. We define part-time work as working less than the aforementioned threshold numbers of hours but more than 0 hours a week.

## 4. Descriptive statistics

Table 1 presents descriptive statistics for the full sample as well as the first and last wave to compare statistics over time. The average age of the sample is 61 years. Almost 15% are between the early and normal retirement ages and about 36% are above the normal retirement age. 28% have a higher education; 76% of the sample are married or have a partner, and 21% report that their health is fair or poor.

The average depression score with 1.8 out of 12 is close to the and clinical threshold for depression based on the EURO-D Score which is 4 or more symptoms. As objective indicators of general physical health, the average number of difficulties in daily activities or in mobility or muscle use seems low (0.03 to 0.39). The average number of chronic diseases is 0.8 out of 9, while 61.5 percent of the sample are overweight and 18.8% are obese. While the average score of the word recall test is just above half of its maximum, the average score of the numeracy test is much higher at 4.2 out of maximum of 5.

35.6% of the sample report working 35 hours or more per week, while 11.2% report working less than 35 hours at the time of the survey.

There are plausible changes in the statistics between the first and last waves. The most notable change is that health status deteriorates across most health indicators. This might also be due to the almost 4 years higher mean age in the last wave.

**Figure 2.**
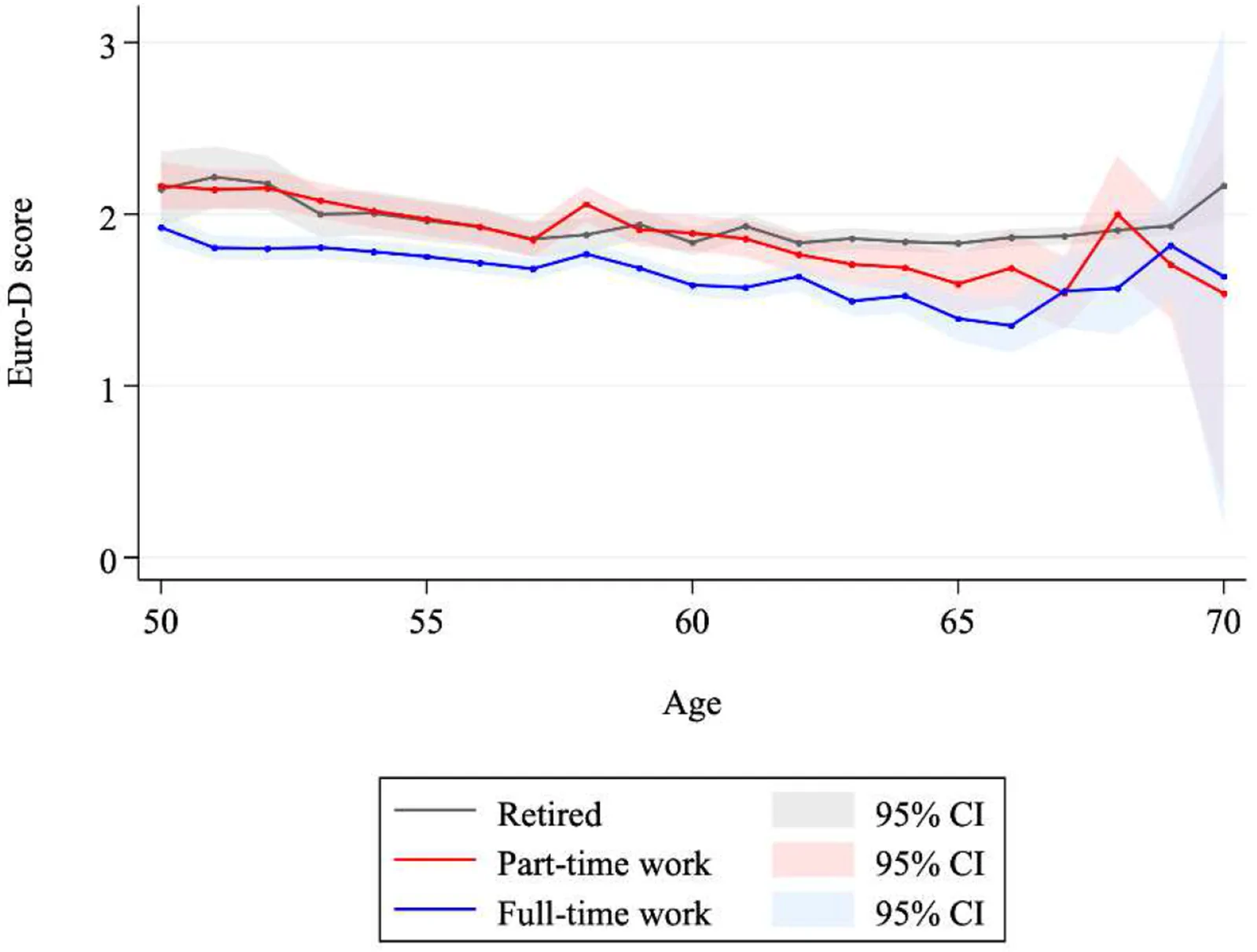
Depression score by age of the respondent among retirees (grey), full-time workers (blue) and part-time workers (red). Mean values and 95% confidence intervals around them.

Figure 2 plots the mean Euro-D score by age of the respondent among retirees, full-time workers and part-time workers. The figure shows that full-time workers have a significantly lower depression score than part-time workers and retirees almost throughout the full age range, although differences are not significantly different from age 62 onwards. The depression score of part-time workers and retired does not seem to be significantly different from each other over the age range.

## 5. Estimation results

Table 2 presents fixed-effects estimates of the Euro-D depression score based on equation (2).^4^ Working part-time increases depressive symptoms by 0.115 points relative to retirement, while full-time work raises the score by 0.135 points. On a scale ranging from 0 (“not depressed”) to 12 (“very depressed”), these effects are modest in absolute size but indicate that employment at older ages, whether part-time or full-time, leads to worse mental health compared to being retired. The similarity in magnitudes of working part-time and full-time suggests that reducing number of work hours does not provide a mental-health advantage over full-time work. It might be that, among older workers, the quality and conditions of work — such as job strain, limited autonomy, or financial necessity — are more important determinants of mental health than hours worked alone. In this interpretation, the rising impact of employment on depressive symptoms likely reflects that individuals who remain in the labour force at older ages often do so under circumstances that are themselves linked to poorer mental health. The slightly larger coefficient for full-time work suggests that higher work intensity can exacerbate stress, but the small difference between part-time and full-time suggests that the underlying mechanism is not simply hours worked but the broader context in which older individuals continue working.

Age enters with a negative linear term and a positive quadratic term, consistent with the well-documented U-shaped pattern in depressive symptoms over the later life course (Blanchflower and Oswald, 2008; Steptoe et al. 2015).^5^

## 6. Conclusion

Depression imposes a considerable economic burden on society and has important consequences for labour-market participation, productivity, and healthcare use (cf. WHO, 2025; König et al., 2021; Krauth et al., 2014). These concerns are particularly salient in ageing societies, where rising retirement ages mean that more individuals remain in the labour force at older ages. As partial-retirement schemes are increasingly used to facilitate longer working lives, it becomes essential to understand how different work arrangements affect mental health.

In this paper, we examined the causal effects of part-time and full-time work on the mental health of older adults in Europe, accounting for unobserved individual heterogeneity that would otherwise bias these estimates. We find that depressive symptoms increase with full-time work, and that part-time work has effects of a similar magnitude. This is an important finding considering the rising prevalence of depressive symptoms among older Europeans and the associated implications for healthcare expenditures and labour-market outcomes (Zhang et al., 2026). Our results are therefore relevant for policymakers considering part-time work as a pathway to extend working lives, as they show that reducing working hours does not mitigate the mental-health burden associated with continued employment at older ages.

Our results provide the first causal evidence on how differences in working hours at older ages — part-time versus full-time employment — affect depressive symptoms. Future research can investigate whether these effects vary across socio-economic and demographic groups, as disparities in mental-health outcomes may lead to heterogeneous responses to changes in work intensity (König et al., 2021). It would also be valuable to explore how institutional settings, job characteristics, and sectoral conditions shape the relationship between work arrangements and mental health, as these factors differ substantially across countries and labour markets (Doan et al., 2025). These analyses can help policymakers assess whether part-time work can serve as an effective and sustainable pathway to extend working lives without exacerbating mental-health burdens.

## Figures and Tables

**Table 1.** Summary statistics for background characteristics and Euro-D Score

	All waves (Mean)	2004 wave (Mean)	2020 wave (Mean)
Age	61.129	59.801	63.578
Under early ret. age	0.492	0.562	0.369
Between early and normal ret. age	0.149	0.139	0.178
Over normal retirement age	0.359	0.298	0.452
Higher education	0.279	0.266	0.36
Married	0.759	0.786	0.753
Female	0.474	0.409	0.509
Depression scale EURO-D (0–12) (avg.)	1.831	1.806	1.801
Full-time worker	0.356	0.425	0.341
Part-time worker	0.112	0.131	0.132
Full-time retiree	0.533	0.444	0.527
Observations	114,277	11,518	7,912
Individuals	42,568	11,518	7,912

Note: Totals may not add due to rounding errors.

**Table 2.** Fixed effects model explaining Euro-D score

	Depression score
Part-time	0.115***
	(0.035)
Full-time	0.135***
	(0.027)
Age	−0.160***
	(0.032)
Age squared	0.001***
	(0.000)
Constant	6.382***
	(0.962)
F test age terms	15.290***
F test employment	13.260***
Observations	81,321
Individuals	39,668

Notes:

1. Linear model with fixed effects.

2. ***, **, * represent statistical significance at the 1%, 5%, and 10% levels, respectively.

3. Standard errors and test statistics are robust to heteroskedasticity and clustering on panel groups.

4. The Euro-D scale measures current depressive symptoms based on 12 items — depressed mood, pessimism, suicidality, guilt, sleep problems, loss of interest, irritability, appetite changes, fatigue, concentration difficulties, reduced enjoyment, and tearfulness. Scores range from 0 (“not depressed”) to 12 (“very depressed”).

**Figure A1.**
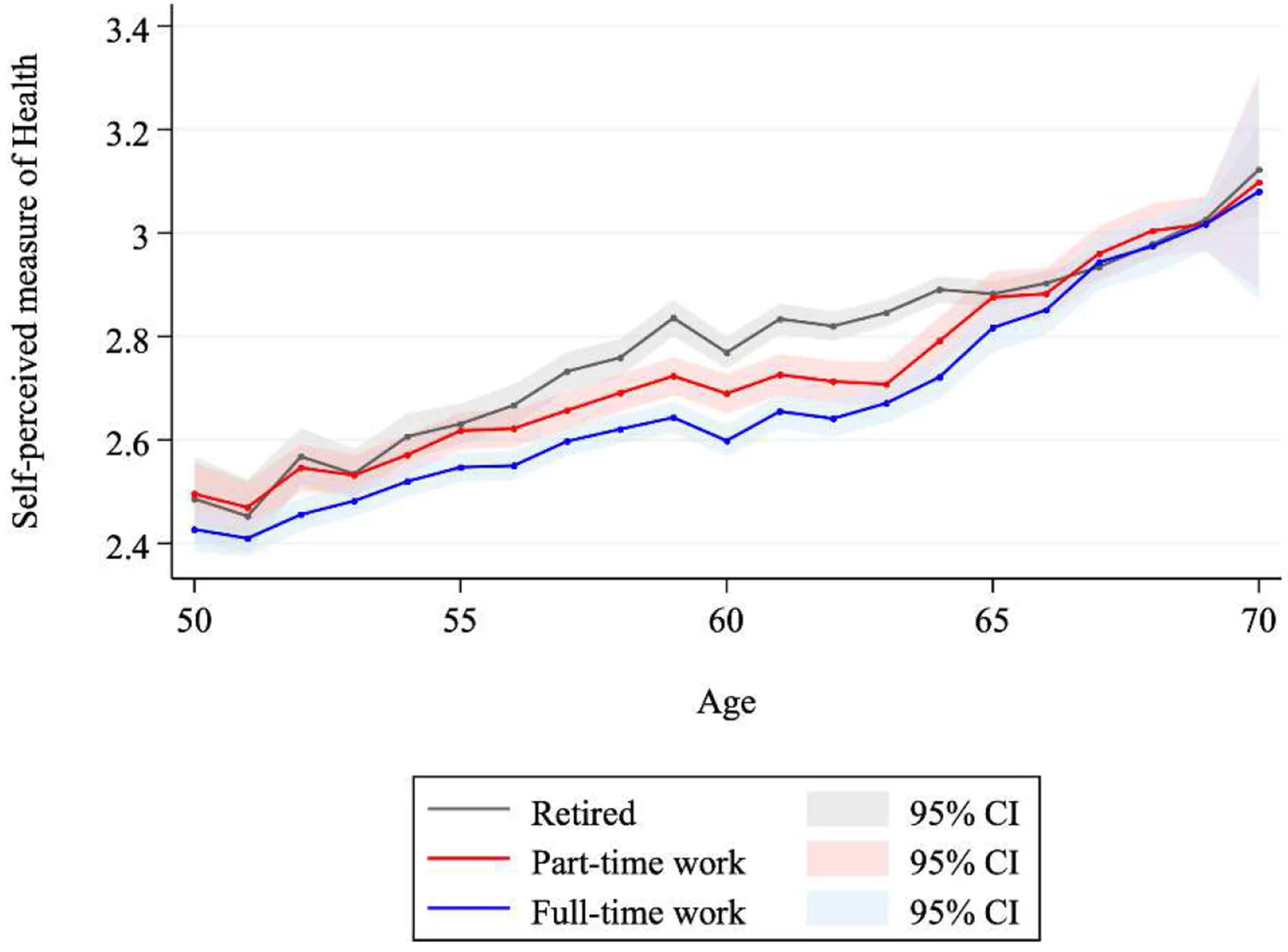

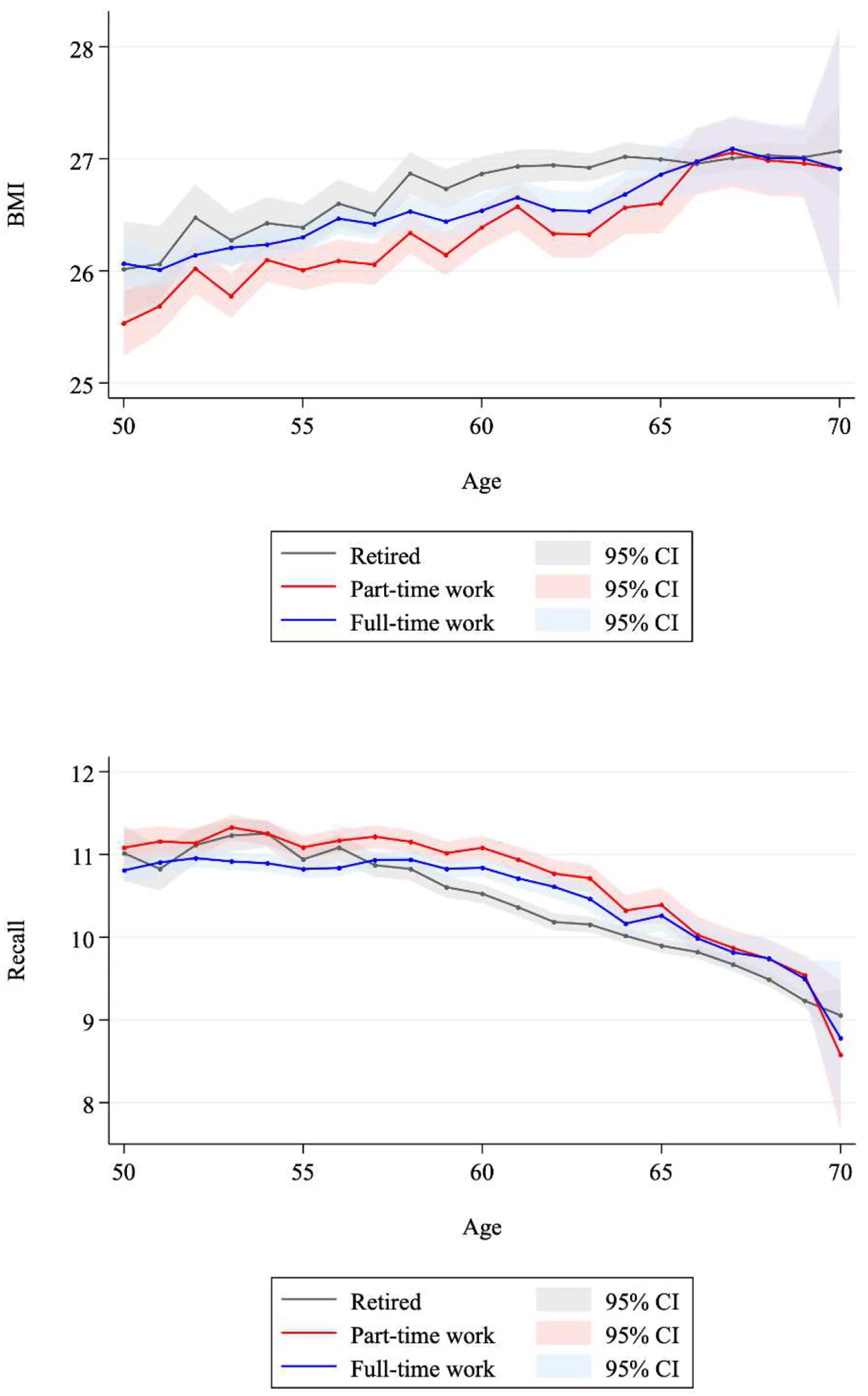

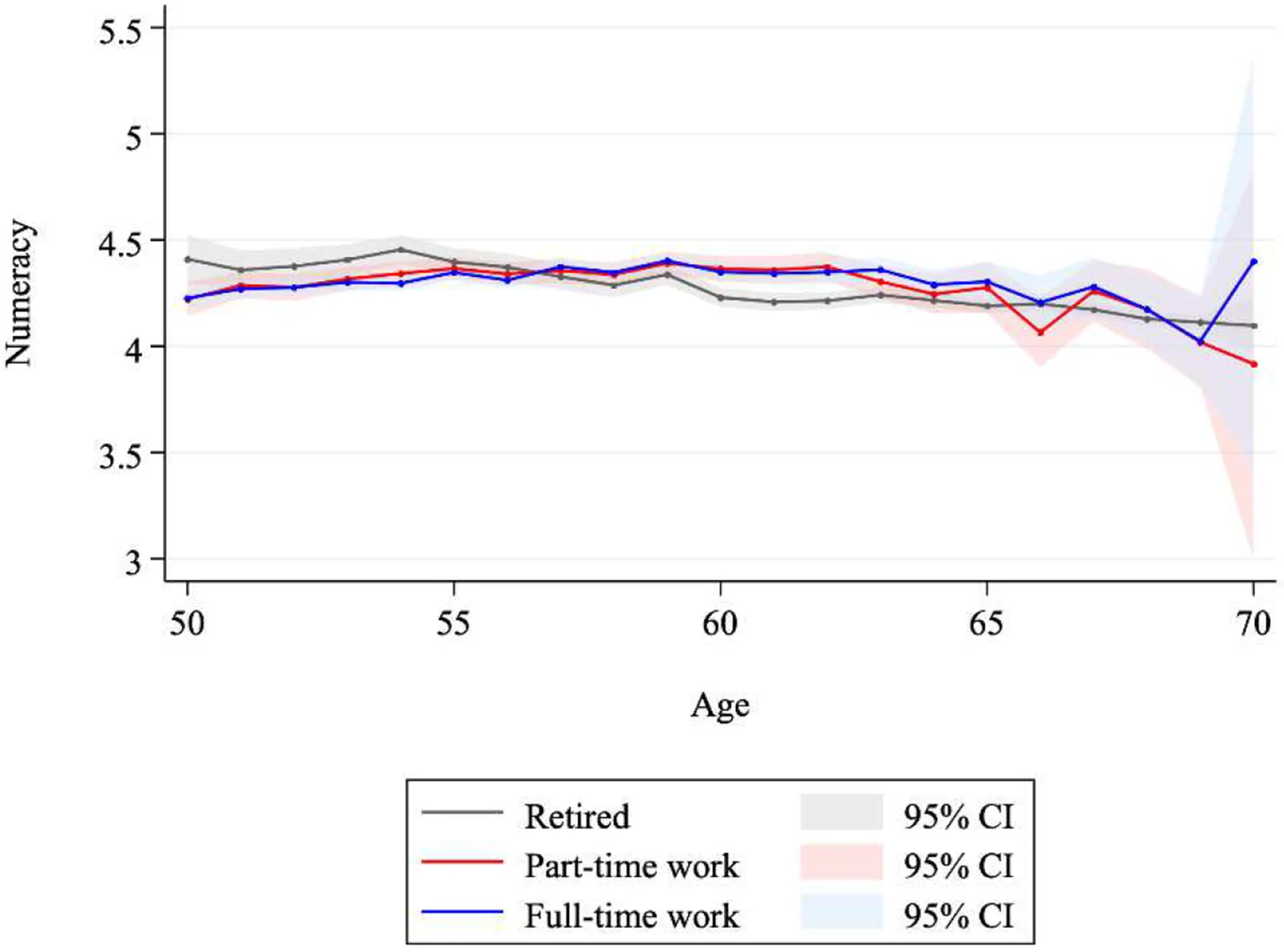
Health measures by age of the respondent among retirees (grey), full-time workers (blue) and part-time workers (red). Mean values 95% confidence intervals around them.

**Figure A2:**
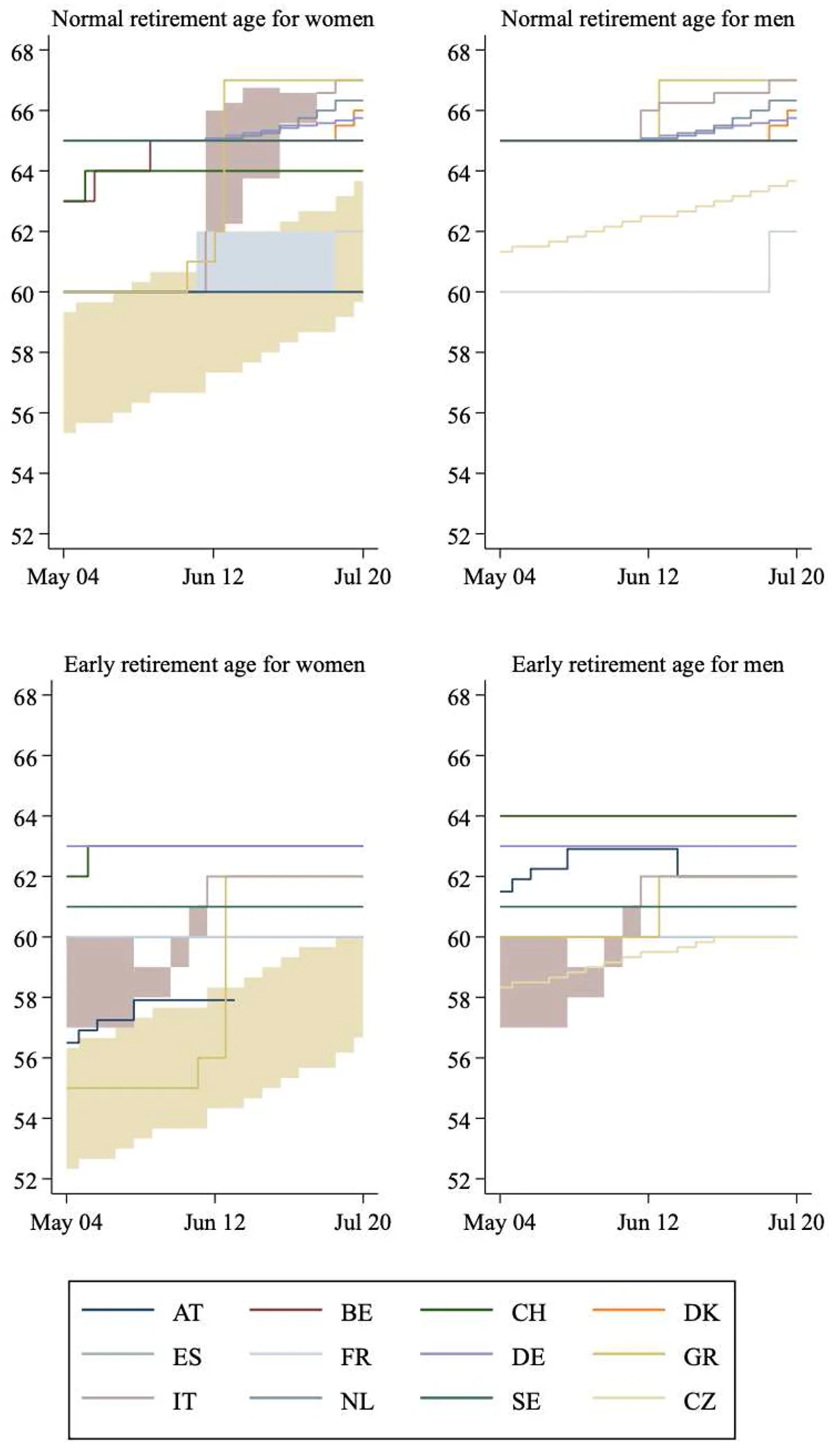
Variation in retirement eligibility ages

**Figure A3.A:**
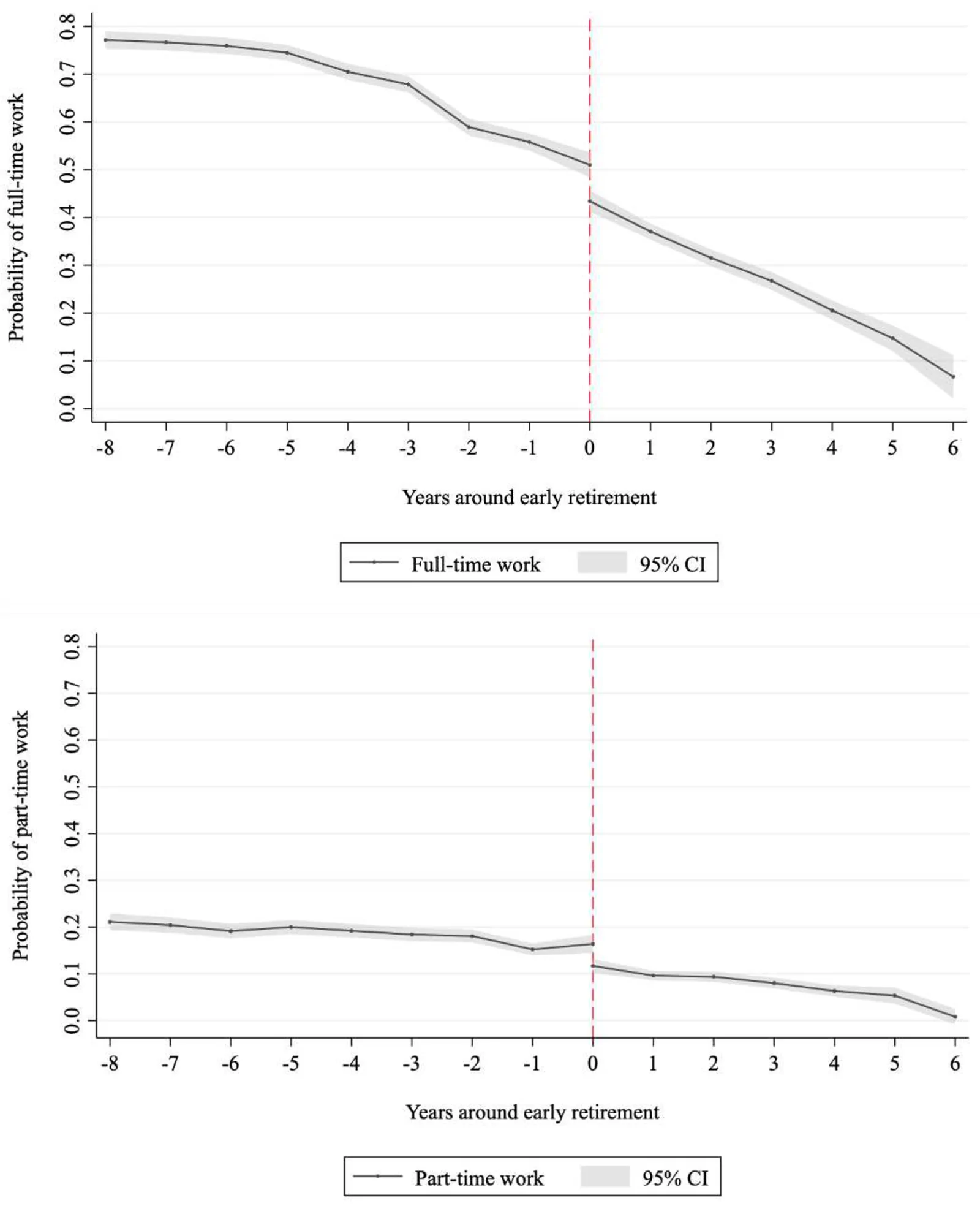
Probability of working full-time (upper panel) and part-time (lower panel) by the years around early retirement age. Mean probabilities and 95% confidence Intervals around them. The figure excludes Denmark and the Netherlands which are presented separately in the appendix.

**Figure A3.B:**
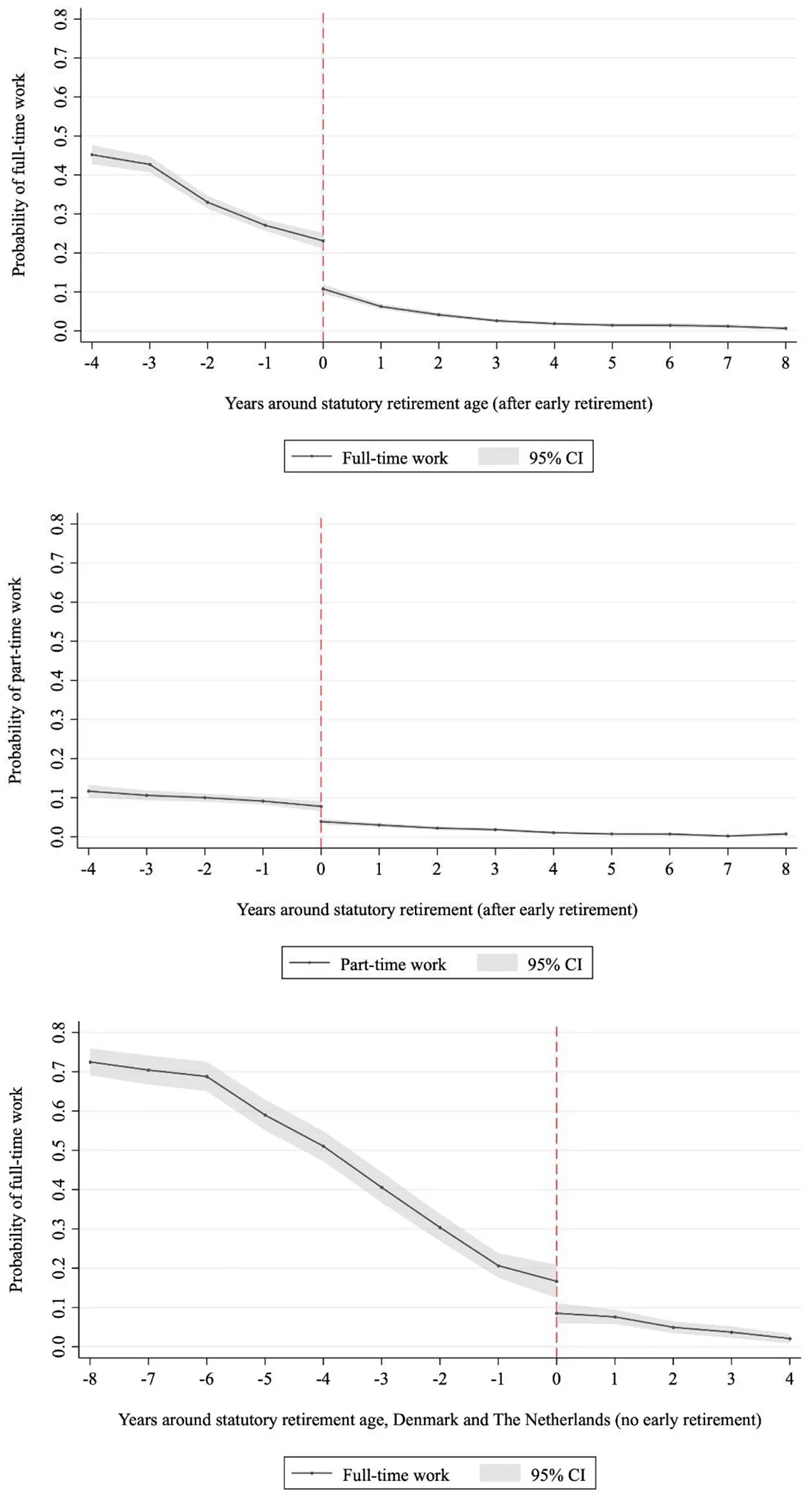

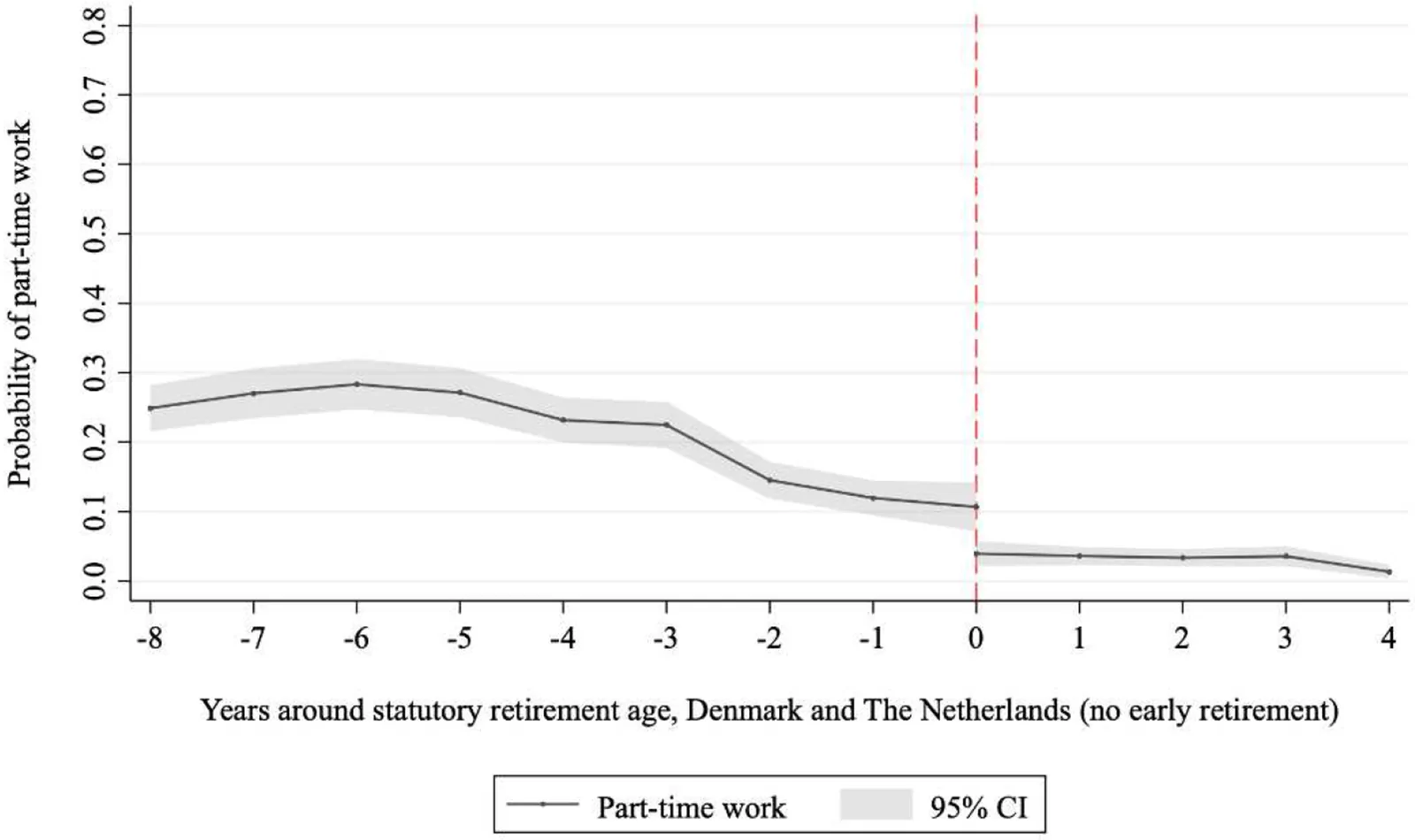
Probability of working full-time (upper panel) and part-time (lower panel) by the years around statutory retirement age. Mean probabilities and 95% confidence Intervals around them.

## Data Availability

This paper uses data from the SHARE study (Survey of Health, Ageing and Retirement in Europe). The SHARE data are available free of charge to the scientific community upon registration and application at the SHARE Research Data Center: https://share-eric.eu/data/. Due to SHARE ERIC’s data protection rules, the authors cannot publicly share the data themselves. This paper uses data from SHARE Waves 1, 2, 4, 5, 6, 7 and 8 (DOIs: 10.6103/SHARE.w1.900, 10.6103/SHARE.w2.900, 10.6103/SHARE.w4.900, 10.6103/SHARE.w5.900, 10.6103/SHARE.w6.900, 10.6103/SHARE.w6.DBS.100, 10.6103/SHARE.w7.900, 10.6103/SHARE.w8.900, 10.6103/SHARE.w8ca.900) see Börsch-Supan et al. (2013) for methodological details. The SHARE data collection has been funded by the European Commission, DG RTD through FP5 (QLK6-CT-2001-00360), FP6 (SHARE-I3: RII-CT-2006-062193, COMPARE: CIT5-CT-2005-028857, SHARELIFE: CIT4-CT-2006-028812), FP7 (SHARE-PREP: GA N°211909, SHARE-LEAP: GA N°227822, SHARE M4: GA N°261982, DASISH: GA N°283646) and Horizon 2020 (SHARE-DEV3: GA N°676536, SHARE-COHESION: GA N°870628, SERISS: GA N°654221, SSHOC: GA N°823782, SHARE-COVID19: GA N°101015924) and by DG Employment, Social Affairs & Inclusion through VS 2015/0195, VS 2016/0135, VS 2018/0285, VS 2019/0332, VS 2020/0313, SHARE-EUCOV: GA N°101052589 and EUCOVII: GA N°101102412. Additional funding from the German Federal Ministry of Research, Technology and Space (01UW1301, 01UW1801, 01UW2202), the Max Planck Society for the Advancement of Science, the U.S. National Institute on Aging (U01_AG09740-13S2, P01_AG005842, P01_AG08291, P30_AG12815, R21_AG025169, Y1-AG-4553-01, IAG_BSR06-11, OGHA_04-064, BSR12-04, R01_AG052527-02, R01_AG056329-02, R01_AG063944, HHSN271201300071C, RAG052527A) and from various national funding sources is gratefully acknowledged (see www.share-eric.eu).

## Footnotes

4 In Section A.2 we present fixed-effects estimates for self-perceived health, BMI, word-recall score, and numeracy. We do not find any significant effects of part-time or full-time employment on these outcomes. It is important to note, however, that the fixed-effects specification is appropriate only for BMI, word recall, and numeracy. In Section A.3 we show that part-time and full-time work decisions are not endogenous to these three outcomes, implying that an IV strategy is not required. By contrast, self-perceived health is endogenous to employment status, and therefore the fixed-effects estimates for that outcome should not be interpreted causally.

5 In Appendix A4, we report estimates from specifications that include linear and cubic polynomials in age. Across these alternative age-flexibility choices, the estimated effects of working part-time and full-time remain largely similar, indicating that our main results are not sensitive to the functional form used to capture age-related health trends.

## Online Appendix

### A1. Descriptive statistics on health outcomes

**Table A1.** Descriptive statistics

	All waves (Mean)	2004 wave (Mean)	2020 wave (Mean)
Self-perceived fair or poor health	0.205	0.176	0.178
Number of ADL limitations (0–5) (avg.)	0.061	0.058	0.062
Number of IADL limitations (0–5) (avg.)	0.032	0.032	0.031
Number of mobility limitations (0–4) (avg.)	0.221	0.212	0.221
Number of difficulties in muscle use (0–4) (avg.)	0.386	0.398	0.402
Number of chronic diseases (0–9) (avg.)	0.844	0.812	0.861
Hospital stay in the previous year	0.108	0.097	0.102
Overweight	0.615	0.605	0.609
Obese	0.188	0.156	0.199
Word recall test score (0–20) (avg.)	10.337	9.123	10.964
Numeracy (0–5) (avg.)	4.242	3.639	4.486
Fluency (0–100) (avg.)	22.387	20.811	23.893
Observations	114,277	11,518	7,912
Individuals	42,568	11,518	7,912

Note: Totals may not add due to rounding errors.

### A2. Fixed effects regressions explaining health outcomes

**Table A2.** Fixed effects model explaining health outcomes

	Self-perceived health	Body mass index	Word recall score	Numeracy
Part-time	0.020	−0.074	−0.021	−0.038*
	(0.017)	(0.046)	(0.058)	(0.021)
Full-time	0.016	0.004	−0.064	−0.016
	(0.013)	(0.033)	(0.045)	(0.017)
Age	0.004	0.366***	0.440***	0.118***
	(0.015)	(0.041)	(0.054)	(0.021)
Age squared	0.000	−0.003***	−0.003***	−0.001***
	(0.000)	(0.000)	(0.000)	(0.000)
Constant	1.798 ***	14.213***	−3.896**	−0.610
	(0.463)	(1.231)	(1.625)	(0.617)
F test age terms	269.440***	137.200***	57.760***	387.880***
F test employment	0.990	1.560	1.060	1.610
Observations	82,393	81,008	81,533	82,131
Individuals	40,030	39,599	39,726	39,965

Notes:

1. Linear model with fixed effects.

2. ***, **, * represent statistical significance at the 1%, 5%, and 10% levels, respectively.

3. Standard errors and test statistics are robust to heteroskedasticity and clustering on panel groups.

4. The Euro-D scale measures current depressive symptoms based on 12 items — depressed mood, pessimism, suicidality, guilt, sleep problems, loss of interest, irritability, appetite changes, fatigue, concentration difficulties, reduced enjoyment, and tearfulness. Scores range from 0 (“not depressed”) to 12 (“very depressed”).

### A3. Instrumental variables as an identification strategy

#### The regression model

In regression equation (2), the parameter of interest β may be biased due to simultaneity. To address this bias, we consider an instrumental-variables (IV) approach. Following Coe and Zamarro (2011) and many other studies examining the causal effects of retirement on health, we use early and statutory retirement eligibility ages as instruments.

Figure A2 illustrates the substantial variation in retirement eligibility ages — across countries, between early and statutory retirement schemes within countries, and between men and women. Table A1 documents the substantial changes in part-time and full-time employment rates at the retirement eligibility thresholds. Figures A3 and A4 present event-study plots pooling all countries, where the event is reaching either the early or statutory retirement age. Both figures reveal pronounced discontinuities in part-time and full-time employment rates at these eligibility ages.

These eligibility thresholds are plausibly correlated with labour supply decisions, as individuals who become eligible for retirement face reduced financial incentives to continue working. At the same time, retirement eligibility ages are unlikely to be correlated with individual health, since they are set institutionally within pension schemes rather than determined by personal characteristics.

The IV estimation proceeds in two stages. In the first stage, we model part-time and full-time working decisions as a function of retirement eligibility using

(4) $$W_{it}=f\left(A_{it}\right)+\text{γ}\cdot I\left(A_{it}>A\right)+{\text{η}}_{i}+{\text{ϵ}}_{it},$$

where f(A_it_) is a polynomial in age, and I(A_it_ > A) is an indicator equal to one if respondent i has passed the relevant retirement eligibility age, either early or statutory. The coefficient γ captures the difference in labour supply between individuals who are not yet eligible for pension benefits and those who are eligible. It therefore measures the change in the probability of working, either part-time or full-time, induced by reaching the eligibility threshold. The term η_i_ denotes unobserved, time-invariant individual heterogeneity, while ϵ_it_ is the idiosyncratic error term, assumed to be exogenous to the model’s explanatory variables.

The first-stage model is used to obtain predicted values of W_it_. Since W_it_ consists of dummy variables, these fitted values represent predicted probabilities of working part-time or full-time. This follows from the fact that equation (5) is estimated as a linear probability model.

The predicted probabilities are then used as instruments in the second-stage equation:

$${\text{Y}}_{\text{it}}=\text{α}+\text{β}\cdot {\hat{\text{W}}}_{\text{it}}+\text{f}\left({\text{A}}_{\text{it}}\right)+{\text{e}}_{\text{i}}+{\text{ε}}_{\text{it}}$$

A key identification requirement is that, since there are two endogenous regressors, the part-time and full-time indicators, the instrument set must contain at least two sources of exogenous variation that shift each work margin independently. In other words, the excluded instruments must be sufficiently strong and sufficiently distinct to separately identify the causal effect of part-time work and the causal effect of full-time work. Without such independent variation, the model risks weak identification or collinearity in the first stage. To address this identification concern, we rely on the weak-instrument diagnostic proposed by Sanderson and Windmeijer (2016). Their F-test is specifically designed for linear IV models with multiple endogenous regressors, providing a way to assess the strength of the excluded instruments for each endogenous variable conditional on the others. This allows us to verify whether the instrument set offers sufficiently strong and independent variation to identify the separate effects of part-time and full-time work.

**Table A3.** Part- and full-time employment rates by retirement eligibility ages

	Full-time worker	Part-time worker	Full-time retiree	N
Under early retirement age	66.17	20.46	13.37	36,555
Between early and normal retirement age	31.54	8.91	59.56	12,262
Over the normal retirement age	3.74	1.87	94.38	33,608
Under early retirement age for partner	55.37	16.08	28.55	35,260
Between early and normal retirement age for partner	27.39	9.20	63.40	8,539
Over the normal retirement age for partner	9.66	4.85	85.49	22,375
Observations	29,313	9,200	43,912	

Notes:

1. P: Married or unmarried partner.

2. The employment status is based on the working hours.

3. Other employment statuses ‘disabled’, ‘not in the labor force’, and ‘unemployed’ are excluded from the analysis.

#### Regression results

Table A3.1 presents the first-stage estimates for working part-time and working full-time relative to retirement, based on equation (4). Respondents’ retirement eligibility ages have significant negative effects on both outcomes. The magnitudes are larger for full-time work than for part-time work, and the effects at the normal retirement age exceed those at the early retirement age. This difference is expected because the normal retirement age typically marks the age at which financial incentives to exit employment become strongest, generating a sharper change in the relative attractiveness of continued work. Partners’ retirement eligibility ages also affect respondents’ labor supply decisions, particularly the partner’s normal retirement eligibility age, although these effects are smaller than those of own eligibility. This asymmetry is consistent with standard labor-supply theory: an individual’s own eligibility directly alters their budget constraint and opportunity cost of working, whereas the partner’s eligibility affects labor supply only indirectly through household income and value of shared leisure time. The F-test of the joint significance of all instruments further confirms that the retirement-eligibility variables are strong predictors of labor-supply choices, providing clear support for the first identifying assumption of instrument relevance.

The Sanderson–Windmeijer F-tests reject the null hypothesis that the excluded instruments fail to provide independent explanatory power for the part-time and full-time first-stage equations at the 10 percent significance level, providing limited support that the retirement-eligibility ages provide sufficiently strong and distinct variation to separately identify the two endogenous work margins.

**Table A3.1.** First-stage regressions explaining probability of working part-time and full-time

	Part-time	Full-time
Between early and normal retirement age	−0.046***	−0.134***
	(0.005)	(0.007)
At or over the normal retirement age	−0.090***	−0.285***
	(0.006)	(0.008)
Between early and normal retirement age (P)	−0.002	−0.041***
	(0.006)	(0.007)
At or over the normal retirement age (P)	−0.021***	−0.053***
	(0.007)	(0.009)
Age	0.027***	−0.031***
	(0.006)	(0.008)
Age squared	0.000***	0.000
	(0.000)	(0.000)
Constant	−0.517***	2.255***
	(0.199)	(0.254)
F test for four instruments	63.780***	366.820***
F test for weak identification	2.340*	2.470*
Observations	66,174	66,174
Individuals	33,153	33,153

Notes:

1. Linear probability model with fixed effects.

2. P: Married or unmarried partner.

3. ***, **, * represent statistical significance at the 1%, 5%, 10% levels, respectively.

4. Weak identification test tests the null hypothesis that the particular endogenous regressor is not identified. This test is based on the Sanderson-Windmeijer F statistic.

5. Standard errors and test statistics are robust to heteroskedasticity and clustering on panel groups.

Table A3.2 presents the second-stage estimates based on equation (5). At the 5 percent significance level, the endogeneity test indicates that part-time and full-time work are endogenous only in the regression for self-perceived health. For the other outcomes, the null that these variables can be treated as exogenous cannot be rejected. This implies that the instrumental-variables strategy is not warranted for those outcomes, and we therefore rely on equation (2) to explain the health outcomes.

**Table A3.2.** Second-stage regressions explaining health outcomes

	Self-perceived health	Body mass index	Word recall score	Numeracy	Depression score
Part-time	−0.099	−1.223	−3.735	0.011	0.268
	(0.892)	(2.169)	(3.213)	(1.201)	(1.763)
Full-time	0.253	0.241	0.769	−0.120	0.229
	(0.284)	(0.680)	(1.041)	(0.385)	(0.567)
Age	−0.014	0.401***	0.591***	0.125**	−0.168**
	(0.038)	(0.091)	(0.137)	(0.053)	(0.077)
Age squared	0.000	−0.003***	−0.005***	−0.001*	0.001**
	(0.000)	(0.001)	(0.001)	(0.000)	(0.001)
Endogeneity (C-stat)	12.689***	0.297	5.329*	1.583	1.593
Overidentification test	5.971*	8.796**	6.764**	13.384***	2.680
F test age terms	124.050***	23.200***	21.880***	38.370***	16.390***
F test employment	15.940***	1.180	5.240*	2.340	7.330**
Observations	51,164	50,111	50416	50892	50281
Individuals	18,159	17,835	17915	18066	17882

Notes:

1. Linear instrumental-variables model with individual fixed effects.

2. Self-perceived health ranges from 1 (very good) to 5 (very bad). Body mass index ranges from 12.5 to 97.8; higher values indicate higher body weight. Word-recall scores range from 0 to 20, with higher values indicating better memory performance. Numeracy ranges from 1 (low) to 5 (high). Depression scores range from 0 to 12, with higher values indicating more severe symptoms.

3. Standard errors are robust to heteroskedasticity and clustered at the individual level.

4. The endogeneity test evaluates the null hypothesis that the specified endogenous regressors can be treated as exogenous. The statistic is computed as the difference between two Sargan–Hansen statistics and is robust to heteroskedasticity and clustering.

5. The overidentification test evaluates the null hypothesis that the excluded instruments are uncorrelated with the structural error term. The statistic is based on the Hansen J-test and is robust to heteroskedasticity and clustering.

6. ***, **, * represent statistical significance at the 1%, 5%, 10% levels, respectively.

### A4. Sensitivity analysis

Our baseline regression in Section 5 employs a quadratic function of age to capture nonlinearities in the health outcome. In Appendix A4, we assess the sensitivity of the results to alternative specifications by estimating models with linear and cubic polynomials in age. Table A4.1 reports the estimates from the linear and cubic specifications and reproduces the baseline quadratic results. The estimated effects of working part-time and full-time are similar across all three age-polynomial specifications, indicating that our findings are not sensitive to the chosen functional form for age.

**Table A4.1.** Fixed effects model explaining Euro-D score: Sensitivity to age polynomials

	Linear age	Quadratic age	Cubic age
Part-time	0.093***	0.115***	0.115***
	(0.034)	(0.035)	(0.035)
Full-time	0.117***	0.135***	0.135***
	(0.027)	(0.027)	(0.028)
Age	0.005**	0.159***	−0.17
	(0.002)	(0.032)	(0.535)
Age squared		0.001***	0.001
		(0.000)	(0.009)
Age cubed			−0.000
			(0.000)
Constant	1.451***	6.382***	6.593
	(0.154)	(0.962)	(10.71)
F test age terms	4.88**	15.29***	10.20***
F test employment	10.06***	13.26***	12.51***
Observations	81,321		
Individuals	39,668		

Our baseline IV regressions employed a quadratic function of age to capture nonlinearities in the health outcome. Here, we assess the sensitivity of the results to using linear and cubic specifications of age. Tables A4.2A and A4.2B report the first-stage estimates for the linear and cubic polynomials and reproduce the estimates based on the baseline quadratic specification. The main finding is that the estimated effects of the instruments become somewhat smaller when a cubic polynomial is used. This pattern is expected, as a more flexible polynomial absorbs part of the explanatory power that would otherwise be attributed to the instruments.

**Table A4.2A.** First-stage regression explaining probability of working part-time

	Linear age	Quadratic age	Cubic age
Between early and normal ret. age	−0.046***	−0.046***	−0.037***
	(0.005)	(0.005)	(0.006)
At or over the normal ret. age	−0.099***	−0.090***	−0.080***
	(0.007)	(0.006)	(0.007)
Bet. early and nor. ret. age (P)	−0.001	−0.002	−0.001
	(0.006)	(0.006)	(0.006)
At or over the normal ret. age (P)	−0.023***	−0.021*	−0.018***
	(0.007)	(0.007)	(0.007)
Age	−0.003***	0.027***	0.564***
	(0.001)	(0.006)	(0.112)
Age squared		−0.000*	−0.009***
		(0.000)	(0.002)
Age cubed			0.000***
			(0.000)
Constant	0.380***	−0.517***	−11.232***
	(0.034)	(0.199)	(2.257)
F test for two age terms		41.590***	
F test for three age terms			28.840***
F test for four instruments	75.780***	63.780***	45.640***
F test for weak identification	3.100**	2.340*	3.310*
Observations	66,174	66,174	66,174
Individuals	33,153	33,153	33,153

**Table A4.2B.** First-stage regression explaining probability of working full-time

	Linear age	Quadratic age	Cubic age
Between early and normal retirement age	−0.134***	−0.134***	−0.074***
	(0.007)	(0.007)	(0.008)
At or over the normal retirement age	−0.283***	−0.285***	−0.226***
	(0.008)	(0.008)	(0.009)
Between early and normal retirement age (P)	−0.041***	−0.041***	−0.032***
	(0.007)	(0.007)	(0.007)
At or over the normal retirement age (P)	−0.053***	−0.053***	−0.038***
	(0.009)	(0.009)	(0.008)
Age	−0.026***	−0.031***	3.446***
	(0.001)	(0.008)	(0.136)
Age squared		0.000	−0.058***
		(0.000)	(0.002)
Age cubed			0.000***
			(0.000)
Constant	2.112***	2.255***	−67.113***
	(0.044)	(0.254)	(2.729)
F test for two age terms		599.530***	
F test for three age terms			516.950***
F test for four instruments	353.180***	366.820***	227.630***
F test for weak identification	3.210**	2.470*	3.710**
Observations	66,174	66,174	66,174
Individuals	33,153	33,153	33,153

Tables A4.3 reports the second-stage estimates for the three polynomial specifications of age. Our main conclusion regarding the endogeneity of work decisions remains unchanged: except for self-perceived health, we do not find evidence that work decisions are endogenous to the health outcomes. This suggests that, in our data, an instrumental-variables strategy is not required to identify the causal effects of part-time and full-time work decisions.

**Table A4.3.** Second-stage regressions explaining health outcomes

	Self-perceived health	Body mass index	Word recall score	Numeracy	Depression score
Linear age					
Part-time	0.188	0.969	−2.627	−0.362	−1.630
	(0.737)	(1.879)	(2.693)	(0.995)	(1.594)
Full-time	0.092	−0.103	1.048	0.117	0.686
	(0.265)	(0.658)	(0.979)	(0.359)	(0.576)
Age	0.032***	0.050***	0.046**	0.043***	0.014
	(0.005)	(0.012)	(0.018)	(0.007)	(0.011)
Endogeneity (C-stat)	6.642**	5.021*	1.423	0.120	1.648
Overidentification (H.J. stat)	2.132	12.397***	0.049	8.092**	6.015**
F test employment terms	9.210***	3.300	1.290	0.160	1.960
Quadratic age					
Part-time	−0.099	−1.223	−3.735	0.011	0.268
	(0.892)	(2.169)	(3.213)	(1.201)	(1.763)
Full-time	0.253	0.241	0.769	−0.120	0.229
	(0.284)	(0.680)	(1.041)	(0.385)	(0.567)
Age	−0.014	0.401***	0.591***	0.125**	−0.168**
	(0.038)	(0.091)	(0.137)	(0.053)	(0.077)
Age squared	0.000	−0.003***	−0.005***	−0.001*	0.001**
	(0.000)	(0.001)	(0.001)	(0.000)	(0.001)
Endogeneity (C-stat)	12.689***	0.297	5.329*	1.583	1.593
Overidentification (H.J. stat)	5.971*	8.796**	6.764**	13.384***	2.680
F test age terms	124.050***	23.200***	21.880***	38.370***	16.390***
F test employment terms	15.940***	1.180	5.240*	2.340	7.330**
Cubic age					
Part-time	−0.796	−0.994	−1.894	0.605	1.037
	(0.783)	(1.725)	(2.442)	(1.003)	(1.438)
Full-time	0.507*	0.151	−0.091	−0.410	−0.051
	(0.273)	(0.591)	(0.873)	(0.356)	(0.511)
Age	−0.262	0.575	3.923*	1.438*	0.044
	(0.621)	(1.333)	(2.010)	(0.800)	(1.185)
Age squared	0.005	−0.006	−0.061*	−0.023*	−0.002
	(0.010)	(0.022)	(0.034)	(0.014)	(0.020)
Age cubed	−0.000	0.000	0.000	0.000	0.000
	(0.000)	(0.000)	(0.000)	(0.000)	(0.000)
Endogeneity (C-stat)	12.317***	0.352	8.038**	4.003	1.363
Overidentification (H.J. stat)	5.196*	8.956**	5.322*	10.070***	2.661
F test age terms	216.120***	66.450***	46.220***	126.580***	15.960***
F test employment terms	10.810***	1.270	9.290***	4.520	4.590
